# IgG4-Related Disease and Synchronous Marginal Zone Lymphoma Evaluated by 99mTc-iFAP SPECT/CT and [ ^18^ F]-FDG PET/CT: A Case Report

**DOI:** 10.1055/s-0045-1810609

**Published:** 2025-07-31

**Authors:** Joel E. Vargas-Ahumada, Miguel A. Corza-Ayala, Rodrigo Hernández-Ramírez, Myrna A. Luna-Gutierrez

**Affiliations:** 1 Nuclear Medicine Department, Instituto Nacional de Ciencias Médicas y Nutrición Salvador Zubirán (INCMNSZ), México City, México; 2 Department of Radioactive Materials, Instituto Nacional de Investigaciones Nucleares (ININ), Ocoyoacac, Mexico

**Keywords:** IgG4-related disease, marginal zone lymphoma, [ ^18^ F]-FDG PET/CT, 99mTc-iFAP SPECT/CT, molecular imaging, FAPI

## Abstract

**Background:**

IgG4-related disease (IgG4-RD) is a chronic fibro-inflammatory condition that can mimic malignancy due to its tumorous lesions. Marginal zone lymphoma (MZL), a low-grade B cell lymphoma, may occasionally arise in the context of chronic immune stimulation. The synchronous occurrence of IgG4-RD and MZL is rare and diagnostically challenging.

**Case Report:**

We report the case of a 39-year-old male with right hemiplegia and aphasia, initially suspected to have a meningioma based on magnetic resonance imaging (MRI) findings. Histopathology following surgical resection revealed features consistent with IgG4-RD. Subsequently, [ ^18^ F]-FDG PET/CT ([ ^18^ F]-fluorodeoxyglucose positron emission tomography/computed tomography) demonstrated a metabolically inactive extra-axial supratentorial lesion, as well as a hypermetabolic triangular consolidation in the left lower lung lobe. Lung biopsy confirmed bronchus-associated lymphoid tissue MZL. Immunohistochemistry supported the dual diagnosis. Complementary 99mTc-iFAP SPECT/CT (single photon emission computed tomography/CT) demonstrated focal uptake in the central nervous system lesion but not in the pulmonary lesion. Multimodal image fusion with brain MRI facilitated precise anatomical and functional correlation.

**Discussion:**

The coexistence of IgG4-RD and MZL raises important considerations regarding shared immunopathogenic mechanisms. Dual molecular imaging with FDG and FAPI (fibroblast activating protein inhibitor)-based radiotracers proved essential in discriminating between inflammatory and neoplastic components of disease, enabling accurate diagnosis and guiding appropriate management.

**Conclusion:**

This case highlights the value of [ ^18^ F]-FDG PET/CT and 99mTc-iFAP SPECT/CT in the evaluation of complex systemic diseases. fibroblast activation protein–targeted imaging may play a key role in the diagnostic workup of IgG4-RD and in distinguishing it from coexisting lymphoproliferative disorders.

## Introduction

IgG4-related disease (IgG4-RD) is a systemic fibro-inflammatory disorder characterized by tissue infiltration of IgG4-positive plasma cells, leading to organ dysfunction. It frequently manifests as tumefactive lesions in multiple organs, including the pancreas, salivary glands, and retroperitoneum. 1 2 3 It can mimic malignancy due to its characteristic tumorous lesions, and the diagnosis is based on typical clinical, serological, pathological, and radiological features. 4 5 Marginal zone lymphoma (MZL), on the other hand, represents a distinct subtype of non-Hodgkin lymphoma arising from B cell proliferation in the marginal zone of lymphoid tissues. 6 While IgG4-RD and MZL are well-recognized as individual entities, their synchronous occurrence is uncommon, presenting unique diagnostic and therapeutic challenges. 7

Molecular imaging has emerged as a pivotal tool in the evaluation of complex systemic diseases, offering functional and metabolic insights that surpass conventional imaging. [ ^18^ F]-FDG PET/CT ([ ^18^ F]-fluorodeoxyglucose positron emission tomography/computed tomography) is widely used for detecting metabolically active lesions in both inflammatory and neoplastic conditions, though its limited specificity can hinder differential diagnosis. 8 More recently, fibroblast activating protein inhibitor (FAPI) PET/CT and in some instances SPECT/CT (single photon emission computed tomography/CT), targeting a protein expressed in stromal and fibrotic tissue, has shown promise in characterizing fibrotic and inflammatory processes with higher specificity. 9 10

In this study, we are the first to report a case of a synchronous IgG4-RD and MZL, a rare and diagnostically challenging overlap, with dual molecular imaging evaluation using [ ^18^ F]FDG PET/CT and 99mTc-iFAP SPECT/CT in differentiating between inflammatory and malignant lesions and refining disease characterization in a complex clinical scenario.

## Case Presentation

A 39-year-old male patient presented with a family history of rheumatoid arthritis and diagnosis of type 2 diabetes mellitus under adequate pharmacological control. There was negative family history of cancer. The patient initially presented with sudden onset of right hemiplegia and aphasia. A brain magnetic resonance imaging (MRI) revealed a probable meningioma in the left frontal region, and prednisone therapy was initiated with partial symptom improvement. Months later, the patient experienced a relapse of right hemiplegia, prompting surgical excision of the lesion. Twenty-four hours after surgery, the patient suffered a cerebrovascular event characterized by bilateral frontal infarction, resulting in visual disturbances as a sequela. Biopsy findings suggested Castleman's disease, and the patient was referred to our institution for further evaluation. Physical examination revealed diminished shoulder elevation and decreased grip strength on the right side, with no other abnormalities noted. Histopathological slides revealed morphological features and immunohistochemical profiles consistent with IgG4-RD. Immunohistochemistry showed positive IgG4 staining in plasma cells (>15 cells per high-power field); positive IgG staining in plasma cells; positive kappa light chain staining (++ in plasma cells); positive lambda light chain staining (+ in plasma cells); CD138 positive in plasma cells: HHV8 negative; CD56 negative. Laboratory findings: immunoglobulin E (IgE): 117; IgG: 14.8; IgM: 0.67; IgA: 5.6; kappa light chains: 22.5; lambda light chains: 20; beta-2 microglobulin: 1.9.

**Fig. 1:**
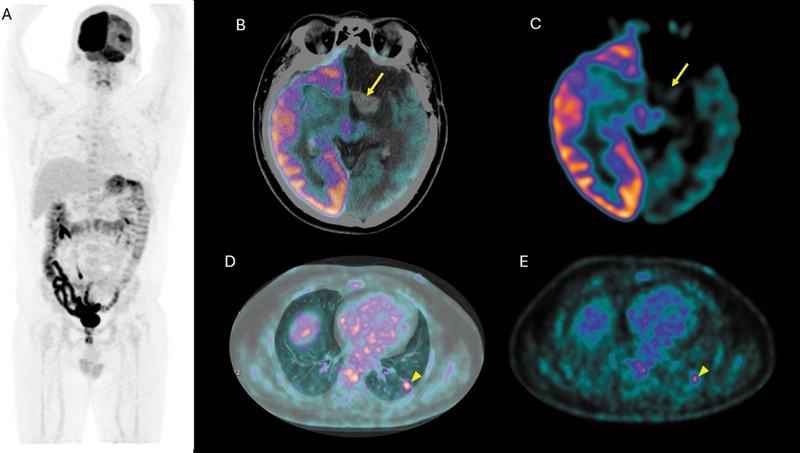
The figures show [ ^18^ F]-FDG PET/CT images: ( **A** ) maximum intensity projection of [ ^18^ F]-FDG PET/CT. Panels ( **B** ) and ( **C** ) demonstrate absence of radiotracer uptake in a hyperdense extra-axial lesion located in the topography of the left thalamus (indicated by arrows). In contrast panels ( **D** ) and ( **E** ) illustrate focal uptake in a nodule identified in the lateral segment of the left lower lobe (indicated by arrowheads). [ ^18^ F]-FDG PET/CT, [ ^18^ F]-fluorodeoxyglucose positron emission tomography/computed tomography.

The patient subsequently underwent an [ ^18^ F]-FDG PET/CT, which revealed a left clinoid extra-axial supratentorial lesion encasing the internal carotid artery, showing contrast enhancement without metabolic activity. Additionally, there was left cerebral hypometabolism and contralateral cerebellar hypometabolism, consistent with crossed cerebellar diaschisis. Furthermore, a solid nodule was observed in the lateral segment of the left lower lobe, associated with focal metabolic activity ( Fig. 1 ). A lung biopsy was performed, with histopathological diagnosis of MZL of bronchus-associated lymphoid tissue. Immunohistochemistry revealed: CD3 reactive, CD20 positive, BCL-2 positive, CD5 positive, cyclin D1 negative, CD43 positive, and Ki67 5%.

**Fig. 2:**
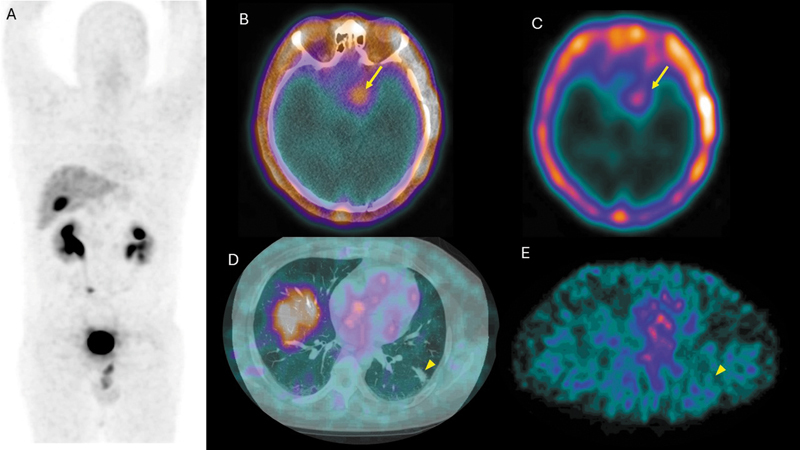
The figures show 99mTc-iFAP SPECT/CT images: ( **A** ) the maximum intensity projection. Panels ( **B** ) and ( **C** ) demonstrate focal radiotracer uptake in a hyperdense extra-axial lesion located in the topography of the left thalamus (indicated by arrows). In contrast, panels ( **D** ) and ( **E** ) illustrate the absence of radiotracer uptake in a solid nodule identified in the lateral segment of the left lower lobe (indicated by arrowheads). SPECT/CT, single photon emission computed tomography/computed tomography.

**Fig. 3:**
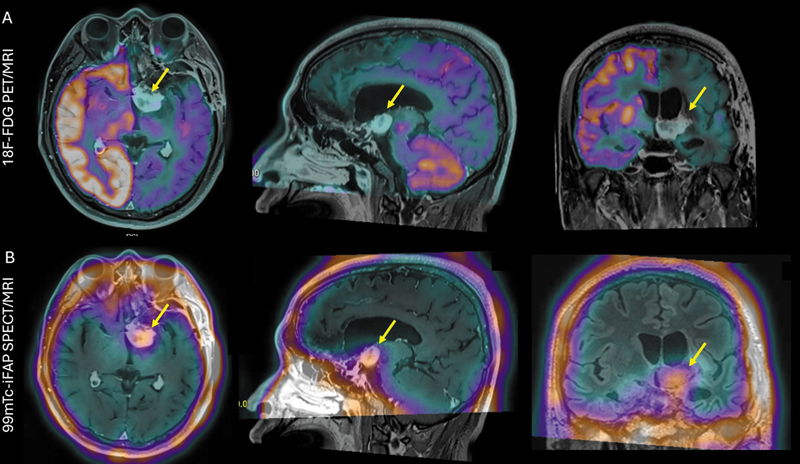
Axial, sagittal, and coronal projection images obtained through fusion of magnetic resonance imaging (MRI) and molecular imaging: ( **A** ) [ ^18^ F]-FDG PET/MRI and ( **B** ) 99mTc-iFAP SPECT/MRI. A hyperdense extra-axial lesion is evident in the topography of the left thalamus (arrows), exhibiting a focal uptake on 99mTc-iFAP SPECT/MRI. No significant hypermetabolism is observed on the corresponding [ ^18^ F]-FDG PET/MRI images. PET, positron emission tomography; SPECT, single photon emission computed tomography

A complementary three-bed SPECT/CT with 99mTc-iFAP was performed, demonstrating focal uptake in the previously described central nervous system lesion ( Fig. 2 ). No radiotracer uptake was observed in the pulmonary lesion. Additionally, image fusion of PET, SPECT, and brain MRI was performed ( Fig. 3 ).

## Discussion

The synchronous occurrence of IgG4-RD and MZL raises intriguing questions about their potential etiological and mechanistic intersection. IgG4-RD is characterized by immune dysregulation, particularly involving T-helper type 2 cells and regulatory T-cells, leading to chronic inflammation. MZL, meanwhile, is associated with chronic antigenic stimulation, often driven by infections or autoimmune conditions. It is plausible that the chronic inflammatory milieu in IgG4-RD may predispose individuals to lymphoproliferative disorders, including MZL. The overlap in clinical and histopathological findings poses significant diagnostic hurdles, necessitating careful differentiation through biopsy and immunophenotyping. 11 12

This case highlights the rare synchronous presentation of the two entities, underscoring the diagnostic complexity when inflammatory and neoplastic processes coexist. The use of dual molecular imaging [ ^18^ F]-FDG PET/CT and 99mTc-iFAP SPECT/CT proved pivotal in delineating the distinct molecular components of this case. [ ^18^ F]-FDG PET/CT remains a cornerstone in lymphoma evaluation, offering high sensitivity for metabolically active lesions, including MZL. 13 In this patient, [ ^18^ F]FDG PET/CT successfully identified the hypermetabolic lesion in the left lung corresponding to MZL, guiding further diagnostic and therapeutic decisions. Conversely, 99mTc-iFAP SPECT/CT, a novel imaging modality targeting fibroblast activation protein (FAP) expressed in fibrotic and stromal tissues, 14 demonstrated selective uptake in the supratentorial lesion correlating with histopathological findings of IgG4-RD. Recent studies have validated FAPI PET/CT utility in characterizing fibrotic and inflammatory lesions with improved sensitivity and specificity over FDG, particularly in IgG4-RD. 15

The complementary nature of these imaging modalities enabled precise functional characterization of both disease entities. This dual-tracer approach exemplifies the evolving role of molecular imaging in complex systemic disorders. Further research is warranted to explore the mechanistic links between IgG4-RD and lymphoid neoplasms and to validate the clinical utility of FAP-targeted imaging in broader inflammatory contexts.

## Conclusion

This case underscores the rare but clinically significant coexistence of IgG4-RD and MZL. The use of dual-tracer molecular imaging with [ ^18^ F]-FDG PET/CT and 99mTc-iFAP SPECT/CT enabled accurate characterization of inflammatory versus neoplastic lesions, facilitating a more comprehensive understanding of the disease spectrum in this patient. [ ^18^ F]-FDG PET/CT successfully identified metabolically active malignant involvement, while 99mTc-iFAP SPECT/CT specifically delineated fibrotic lesions associated with IgG4-RD. This multimodal imaging approach demonstrated the potential to guide differential diagnosis and therapeutic strategy in patients presenting with complex, overlapping systemic conditions. Further studies are warranted to validate the utility of FAP-targeted imaging in inflammatory and lymphoproliferative disorders.
